# Bi-temporal processing in music notation reading: a theory linking prediction, memory, and automaticity

**DOI:** 10.3389/fcogn.2025.1689600

**Published:** 2026-02-06

**Authors:** Karen L. Heath

**Affiliations:** Melbourne Conservatorium of Music, The University of Melbourne, Melbourne, VIC, Australia

**Keywords:** cognition, dual processing, memory, music notation, music reading, performance, prospective planning

## Abstract

Reading music notation requires musicians to extract and interpret visual information in real time while simultaneously anticipating future performance actions. This dual engagement, in which one acts in the present while processing material to be performed in the future, suggests that music reading relies on a bi-temporal cognitive architecture. Grounded in this premise, this theoretical paper develops a model that integrates Hebbian learning and automaticity as core mechanisms supporting the simultaneous perceptual and anticipatory demands of notation-based music performance. A systematic review of neuroimaging studies involving music-reading tasks was conducted to evaluate current evidence on the neural correlates of notation processing. The results of the review showed that music reading engaged distributed cortical and subcortical networks, including regions commonly implicated in text reading, and recruited auditory-motor integration systems essential for music performance. However, most studies isolated single parameters of notation (e.g., pitch identification), thereby limiting ecological validity and constraining interpretations of how musicians process in real-world contexts that require concurrent multi-parameter integration. Complementary research on cognitive prediction, sensorimotor coupling, and perceptual-motor learning demonstrates that musicians employ a dual-pathway system of immediate perception and forward prediction, shaped by Hebbian synaptic strengthening and the development of automaticity through repeated procedural engagement. Synthesizing these findings, this article proposes a bi-temporal cognitive model of music-notation processing that accounts for dynamic interplay between associative learning, predictive processing, and automated motor execution. The implications of this model for cognitive theory and music pedagogy are discussed, with recommendations for empirical approaches to test the bi-temporal framework and advance understanding of real-time cognitive coordination in music performance.

## 1 Introduction

Reading music notation is a fundamental skill for professional musicians who work in orchestral, ensemble, educational, and sessional performance contexts. For professional and/or highly skilled amateur musicians who perform using music notation, musical outcomes arise from the simultaneous processing of auditory and visual information, as well as motoric actions (Bigand et al., 2000; Gaser and Gottfried Schlaug, 2003; Ho et al., 2003; Zimmerman and Lahav, 2012). The notion that musical performance encompasses a range of different functions, perception modalities, and physiological outputs is extensively supported in the literature. Research focused on music in psychological, neurological, and physiological contexts points to significant complexities involved with cognition (Schön and Besson, 2002; Gunter et al., 2003; Stewart, 2005a,b; Sluming et al., 2007), brain plasticity (Stewart et al., 2003a; Stewart, 2005a), memory (Simoens and Tervaniemi, 2013), emotion (Jäncke, 2012; Koshimori, 2018; Schubert, 2013), spatial mapping (Stewart, 2005a), and somatosensory perceptions (Brancucci and San Martini, 1999, 2003; Meister et al., 2004; Brancucci et al., 2005; Wong and Gauthier, 2010b).

### 1.1 Music-notation reading as a cognitive subdomain

In this article, reading music notation is positioned as a specific subdomain of musical cognition operating at the interface of perception and performance. For analytic purposes, the component processes of reading music are classified as follows: (a) visual-symbolic decoding, in which elements found in notated scores are mapped onto abstract pitch, rhythmic, and structural representations; (b) audiative-predictive mapping, where notation is transformed into sonic representations, perceived as mental imagery for sounds yet to be performed; and (c) sensorimotor execution, in which predicted events are realized through motor functions. In this sense, we can see that reading and interpreting music notation is not an isolated skill but rather a notation-based entry point into a larger performance system that coordinates perceptual, predictive, and motor processes within a bi-temporal architecture. This frame of reference allows for both present-focused sensorimotor execution and future-oriented audiative–predictive processing, both anchored in ongoing visual–symbolic decoding.

To explore the cognitive processes responsible for interpreting music notation, we must first acknowledge the multivariate, simultaneous events involved in performing and reconcile them with current viewpoints on multitasking. It is now well-understood that the human brain is unable to consciously make decisions about multiple events simultaneously or hold more than one novel thought at a time (Gazzaley and Rosen, 2016; Hari, 2022). How, then, do we account for the multiple, concurrent, and bi-temporal processing of music notation, aural stimuli, motor action responses, anticipatory sensorimotor planning, and the ensuing musical outcomes? Even just the act of performing motor functions requires continuous updating by sensory systems, using information derived from external stimuli (Kandel et al., 2014).

In consideration of possible explanations for these phenomena, this paper explores the roles that *Hebbian learning* and *automaticity* play in reading music notation and hypothesizes a theory of cognitive processing that incorporates a neural basis for learning and memory formation and the ability to operate in two time zones. The theoretical framework presented in this article is based on the premise that cognition operates in different temporal horizons in which past information is accessed while future actions are anticipated (Schacter et al., 2007, 2017; Tulving, 2002). Following this, the argument is presented that musicians with high-level skills in reading music notation process information in a dual-stimulus paradigm that encompasses both visual and aural categories, resulting in a unified, cohesive musical output.

This paper commences with a summary of frameworks, theories, and concepts that underpin the theory presented for consideration. The second section reviews the literature and evaluates previously proposed hypotheses, theories, and current perspectives related to the neural bases of reading musical notation. Included in this section is a systematic review of brain imaging studies that incorporate music notation reading. A discussion section follows in which the theory central to this paper is presented in full, along with suggestions for testing within the framework of current knowledge. Concluding remarks detail possible implications of integrating a bi-temporal component in current understandings of cognitive processing when reading musical notation. Further investigations in this area could offer a critical contribution to understanding neuronal pathway operations during music reading, with potential implications for music education as well as other domains of learning.

### 1.2 Aims and research questions

The central aim of this study is to present a theory on cognitive mechanisms underlying the processing demands of reading musical notation. Emphasis is given to Hebbian learning and automaticity to support the notion of simultaneous perception and prediction in performance. This inquiry responds to persistent limitations in existing empirical work, which has largely examined isolated parameters of notation such as basic pitch identification or simple rhythmic units. This mode of inquiry, which individuates the various task parameters of playing music from notation, contrasts with the reality experienced by musicians when playing from notation, which necessitates the concurrent processing of numerous visual stimuli (Bigand et al., 2000; Gaser and Gottfried Schlaug, 2003; Ho et al., 2003; Zimmerman and Lahav, 2012). The outcome of synthesizing neuroimaging evidence with broader cognitive research on prediction, cognitive load theory (Sweller, 2011), sensorimotor coupling, and perceptual-motor learning (Schön and Besson, 2002; Gunter et al., 2003; Stewart, 2005a,b; Sluming et al., 2007), is a comprehensive bi-temporal model of music notation processing.

To guide the inquiry and subsequent development of a bi-temporal model of music notation reading, the following research questions were posited:

- What neural systems are consistently implicated in real-time notation reading, and how do these compare to those recruited for text processing and auditory-motor integration?
- How do Hebbian learning mechanisms and automaticity contribute to musicians' capacity to engage in simultaneous present-focused perception and future-oriented prediction?
- To what extent does current literature support or constrain a theoretical account of bi-temporal cognitive architecture in music performance?
- What methodological approaches are required to empirically test this proposed model and more accurately capture dual-pathway perceptual-predictive processing in ecologically valid contexts?

## 2 The neural architecture of music notation reading

To clarify the central thesis of this paper, reading music is classified into three processes: visual-symbolic decoding, audiative-predictive mapping, and sensorimotor execution. Each of these components aligns with specific neural structures. The first component, the visual-symbolic decoding process, involves the occipito-temporal visual system extracting the orthographic features of notation (e.g., notes, clefs, accidentals, etc.) and mapping them onto pitch-rhythm representations. This process depends on ventral visual pathways, including areas that show expertise-related tuning (Bouhali et al., 2017; Proverbio et al., 2024). The second component, audiative-predictive mapping, is carried out by transforming visual information into internally generated auditory imagery, or audiation (Gordon, 1999, 2011), along with predictive models of upcoming sound events. This engages predictive planning mechanisms supported by fronto-parietal systems, including working memory (WM) buffers and the dorsal attention network (DAN), aiding future-focused planning (Palmer and van de Sande, 1993; Wolff et al., 2022). The final component, sensorimotor execution, involves translating predicted musical events into motor programs via premotor, motor, cerebellar, and basal ganglia circuits. These same systems facilitate proceduralization and automaticity (Ashby and Crossley, 2012; Bangert et al., 2006). Central to this paper is the idea that these components operate on two time scales: one governs the real-time execution of previously read material, and the other operates in a predictive mode, where visual information is processed and mapped onto various aspects of music reading.

### 2.1 The musician's process: performance in a bi-temporal paradigm

When a musician has practiced extensively to prepare for a performance, successful musical outcomes are often attributed to muscle memory or to the performer being well-rehearsed. This *a priori* assessment provides a satisfactory explanation of how musicians can carry out numerous actions simultaneously while thinking several steps ahead—that is, until we inquire into the cognitive processing necessary for musicians to sightread, i.e., to play from sheet music without any rehearsal or preparation whatsoever. Muscle memory through repetitive practice can no longer be considered the underlying reason, at least not without some interrogation into the different conditions of performance, such as the level of difficulty, familiarity with the patterns embedded within the music, and the overall predictability of the notation being performed.

Mastering a musical instrument involves the acquisition of numerous skills, some of which are refined to the point of automaticity. Research indicates that varying degrees of automaticity are evident in domains such as language acquisition and mathematics (Jeon and Friederici, 2015). However, because music performance has multiple components, there is a distinction between the operations of music and other activities, with potentially numerous parallel memory retrieval systems operating at varying levels of automaticity (Heath, 2025). Additionally, automaticity occurs within the confines of a strict temporal structure as an essential component of music performance (Wakita, 2016). Within the boundaries of time, a high-level professional musician can typically perform repertoire with little need for conscious control over certain aspects of their playing; instead, a series of instinctive, reflexive actions seems to take place, even when performing novel material from music notation.

The element of time is a crucial aspect in music performance. For one, music is an art form grounded in temporality. Secondly, the prospective planning involved in performing from notation at a high level of competency requires a musician to anticipate future actions while executing multiple tasks simultaneously (Palmer and Drake, 1997). This suggests that dual processing may occur across a range of brain regions, operating in two distinct time zones. In a review of the literature (Lewis and Miall, 2003), evidence is presented for separate neural timing systems, in which specific tasks utilize different systems for categorizing temporality. Here, “automatic” actions relied on circuitry within the motor system, whereas time-based actions required conscious control, actualized in the prefrontal and parietal regions (Lewis and Miall, 2003). More recent research highlights a distinction between time in terms of cognitive perception, defined as *prospective timing* for events that begin in the present and end in the future, and *retrospective timing—*events that began in the past and are to end in the past or the present (Tsao et al., 2022). Here, we observe a connection to how musicians read music as they read ahead, conceptualizing future actions while performing previously read notation in the present moment.

With evidence that the brain can encode temporal information over spans ranging from 1 s to several minutes (Tsao et al., 2022), the notion of dual processing is further supported by Høffding (2014) in a phenomenological study of performance as experienced by musicians. In this study, which situates phenomenological data illustratively rather than as empirical evidence, one of the participants describes the operation of performing music as “two tracks running” in which “an awareness of what you are doing and an awareness of what you would like to do…” (Høffding, 2014, p. 65). For musicians, dual operations through prospective and retrospective planning, within the constraints of time, are fundamental to performance.

The initial process of acquiring any skill begins with focused attention and engagement, facilitated by executive functioning and working memory (WM). According to Sachs and colleagues, multiple sensorimotor processes associated with performing music are understood to be operationalized by these areas of cognition (Sachs et al., 2017). As a cognitive processing action, executive functioning encompasses decision-making, judgment, evaluation, and planning (Gazzaley and Rosen, 2016). WM is the temporary storage of information that allows for planning, responding, comprehending, and decision-making (Baddeley and Hitch, 1974; George and Coch, 2011; Schulze and Koelsch, 2012). Although music performance via notation requires a broad engagement of different brain areas simultaneously (Lee et al., 2007; Olivers and Eimer, 2010; Sala and Gobet, 2017; Schulze and Koelsch, 2012; Silverman, 2010; Yeşil and Nal, 2017; Yurgil et al., 2020), research suggests that performing multiple tasks simultaneously—such as the array of actions required to play music—presents significant limitations because the human brain can only attend to one conscious, or deliberate, thought at a time (Gazzaley and Rosen, 2016; Olivers and Meeter, 2008). A convergence of literature relating to attention through mechanisms such as working memory and executive functioning, which neurobiologically operate within the dorsolateral prefrontal cortex (DLPFC), has determined that attempting to engage in numerous actions at once drastically depletes the fidelity of focus and concentration (Gazzaley and Rosen, 2016; Wieth and Burns, 2014). In essence, what was once referred to as *multitasking* is now considered an action that significantly reduces accuracy, leading to *cognitive overload, divided attention*, and frequent *task switching* (Gazzaley and Rosen, 2016; Strobach et al., 2018; Wieth and Burns, 2014).

To distinguish the tasks that a musician is required to undertake simultaneously while also in a state of prospective planning, a brief list of actions might include the following: (a) *audiation*, the process of conceptualizing and hearing music in one's mind when reading musical notation (Gordon, 2011); (b) plotting out technical considerations in advance, such as bowing patterns, finger placements, and articulation; and (c) accurately playing, in real-time, music notation that had been read only moments earlier. If we accept that a musician performs these three discrete tasks simultaneously, we may conclude that there is a set of cognitive processes controlling each task, each sufficiently automated to enable an immediate response to aural and visual stimuli. After the process of working memory and executive function has enabled the musician to learn materials and skills to a sufficient level of fluency, cognitive processing is offloaded to motor-based systems without the involvement of the prefrontal cortex (PFC; Chein and Schneider, 2005; Kelly and Garavan, 2005). Supporting this premise is the theory of automaticity as applied to performing music from notation stimuli, similar to that of reading text.

### 2.2 Neural architecture: attentional and executive networks

Research in cognitive neuroscience demonstrates that executive function, working memory, and attentional control are not reducible to a single cortical region. Instead, they emerge from coordinated activity across large-scale networks, including the Executive Control Network (ECN), the Dorsal Attention Network (DAN), and the Ventral Attention Network (VAN). During music-notation reading, these networks support shifting between present-focused motor execution and future-focused perceptual prediction, enabling the bi-temporal processing proposed in this model. In this model, executive and attentional networks are not treated as independent causal agents, but as interacting systems that enable or constrain bi-temporal processing. While dorsolateral prefrontal cortex (DLPFC) recruitment has been observed in early stages of learning, fluent performance increasingly relies on distributed network interactions rather than isolated regional activation.

### 2.3 Automaticity and reading musical notation

In contrast to working memory and executive functioning, automaticity mostly bypasses the PFC as memory is instead retrieved in a non-conscious, immediate manner. Two forms of automaticity are distinguished, early-stage and procedural, and both are foundational in circumventing the PFC in accessing memory and actions. Bypassing the PFC helps to prevent cognitive overload from multitasking or task switching, thereby enabling efficient, effortless performance (Servant et al., 2018).

Automaticity can only be achieved after initial engagement with WM and executive functioning; practice and repetition of skills or information initially learned through PFC mechanisms will move toward long-term memory storage (Servant et al., 2018). Moreover, repetitive practice substantially increases the speed and accuracy of automatic content retrieval (Wilkins and Rawson, 2011). Musicians often exhibit degrees of automaticity when performing, with studies revealing reduced activity in the dorsolateral prefrontal cortex during such instances (Tan et al., 2024). This phenomenon is known as the “transient hypofrontality hypothesis” (Dietrich, 2004). Instead of the PFC being activated while performing, posterior areas of the brain become more operational, with regions such as Broca's area showing more activity (Jeon and Friederici, 2015; Liao et al., 2024a).

#### 2.3.1 Early-stage automaticity: instance-based retrieval

Early automaticity emerges when repeated exposure allows specific notational patterns to be retrieved as instances (Logan, 1998). This type of automaticity remains partly dependent on fronto-parietal networks and is sensitive to task complexity. In notation reading, early automaticity supports rapid recognition of familiar rhythmic or scalar patterns.

#### 2.3.2 Procedural automaticity: sensorimotor integration

With extended deliberate practice (Ericsson, 2008), automaticity transitions into proceduralized sensorimotor routines consolidated in cortico-striatal–cerebellar circuits (Ashby et al., 2010). This later-stage automaticity enables parallel processing, freeing predictive systems to operate ahead of real-time performance demands. Procedural automaticity is the form most relevant to bi-temporal music reading. With repetitive practice being the foundation of much music pedagogy (Persson, 1996; Stambaugh, 2011), this time-tested method is crucial for achieving high levels of automaticity in music performance. To understand how repetitive practice fosters automaticity, we now examine the principles of Hebbian learning.

### 2.4 Hebbian learning and automaticity

Hebbian learning theory posits that concurrent neural activity results in lasting synaptic strengthening (Hebb, 1949), which partially explains how motor skill development in instrumental music performance is learned to a point of automaticity. Known also by the phrase “neurons that fire together, wire together,” coined by Carla Schatz of Harvard University to describe Hebb's explanation of his theory (Collins, 2017), Hebbian learning is independent of any feedback-related synaptic strengthening, instead developing more slowly, over time. An example is procedural practice, which requires repetition to establish and then consolidate the necessary skills (Ashby et al., 2007), a common preparatory approach for musicians.

Hebbian learning, which essentially describes a mode of brain plasticity, may be contextualized in music as follows: when a musician practices reading notation and playing, the simultaneous activation of visual neurons (responding to the notes), motor neurons (controlling finger movements), and auditory neurons (responding to the sounds or imagined pitches) causes the affected neural circuits to strengthen their connections. Over time, these repeated associations forge a tight sensorimotor linkage between seeing a note and executing the corresponding action. Cognitive neuroscience frameworks describe this as a form of *common coding* (Prinz, 1997) between perception and action: a joint representation emerges when specific actions are repeatedly paired with specific sensory events. In music, a given note on the staff becomes inextricably associated with the finger movement and the sound that note produces. The result is that perceiving the stimulus (the written note) automatically activates the motor program to play it and the auditory representation of its sound.

Hebbian plasticity explains how reading notation becomes ingrained at the neural level. Units of neurons, or cell assemblies, form new associations to create “chunks” of information (Lörch et al., 2023). Chunking plays a foundational role in the formation of procedural routines in music learning. Research in Neurologic Music Therapy (Thaut et al., 2015) demonstrates that rhythmic and melodic grouping enhances temporal prediction, reduces working-memory load, and stabilizes motor sequencing. Incorporating this perspective strengthens the theoretical link between Hebbian learning and the emergence of multi-note “units” that musicians process as single functional entities. Each rehearsal that links a note on the page with a finger movement and, if a musician is using audiation, a relative understanding of pitch spatiality, reinforces the synaptic connections among the corresponding visual, auditory, and motor neurons. Eventually, these neural pathways become so strong that the presence of one element (e.g., a note written on a staff) may be sufficient to trigger the entire synaptic connection. When this occurs, the optimal outcome for performance is facilitated, allowing the musician to perform in a state of automaticity. Because automaticity is generally understood as a process that allows actions to be performed in parallel and without attention (Schneider and Shiffrin, 1977), fewer mistakes are made. Automaticity also enables musicians to respond to unexpected stimuli during a performance or to focus on elements of stagecraft to enhance audience engagement. Furthermore, chunking mechanisms directly support the bi-temporal model by allowing larger perceptual units to be prepared in Time Zone 1 while Time Zone 2 handles execution.

## 3 Neural foundations of music-notation processing: evidence and limitations

To lay a foundation for understanding the present discussion, we must first examine the current perspectives, research, and theories related to the neural basis of interpreting music notation and related understandings in the domains of neuroscience and psychology. This will be followed by a systematic review of brain imaging studies that incorporate aspects of reading musical notation. Through this review, I highlight that for the most part, the foci within the stimulus materials for musician-participants of these studies do not replicate real-world applications of reading and interpreting musical notation. A dissonance exists between the data presented in some of these studies and the assumptions made about the neural bases of reading music in real performance contexts. This dissonance, in part, offers a rationale for the theory presented in this article, as it is grounded in the common, everyday experiences of performing in a musical context.

### 3.1 Neural mechanisms supporting visual, predictive, and motor pathways

When engaged in music performance, cognitive processes relating to memory and motor function are fundamentally interconnected (Bigand et al., 2000; Ho et al., 2003; Zimmerman and Lahav, 2012). The various roles of motor function components are well-understood in the context of learning music (Edwards and Hodges, 2008; Wilson, 1986). Motor function learning is linked to procedural memory (Ashby et al., 2003), with automaticity considered to be an outcome of deliberate music practice (Ashby and Crossley, 2012). These include the relationships between large and small muscles, coordination when playing an instrument, proprioception—the physical manifestation of rhythm—and the execution of motor function after conceptualizing mental plans for action (Palmer and Meyer, 2000; Sidnell, 1986).

The interpretation of music notation in the context of traditional Western art music relies on the visual system^1^. As the eyes scan the score, visual cortices process the basic features of the symbols (lines, spaces, and note heads). In musically literate individuals, higher-order visual areas develop specialized responses to notation. Functional magnetic resonance imaging (fMRI) studies have shown that reading music engages the occipito-temporal cortex, particularly in the right hemisphere, in a manner distinct from reading text or numbers (Schön et al., 2002).

Saccades, the rapid eye movements that occur between fixations, are a crucial component of visual information processing during sight reading. In an influential study, Goolsby demonstrated that highly proficient pianists did not confine their gaze to individual notes but instead employed dynamic saccadic movements across the score, suggesting that their processing extended beyond immediate visual input (Goolsby, 1994). By contrast, less experienced readers displayed longer fixations on discrete notes, suggesting a more localized, effortful mode of visual engagement. These findings highlight significant differences in how visual stimuli are processed as a function of skill level, with advanced performers relying less on conscious decoding and more on anticipatory processing.

Subsequent research has reinforced this view. Waters, Townsend, and Underwood found that skilled pianists exhibited shorter fixation durations and a wider perceptual span compared to novices, enabling them to preview and integrate upcoming material with minimal disruption to performance (Waters et al., 1998). Similarly, Furneaux and Land observed that expert pianists made more forward-directed saccades, reflecting a predictive mechanism that supports real-time motor execution (Furneaux and Land, 2000). More recently, Penttinen et al. demonstrated that proficient sight-readers engaged in anticipatory fixations, allowing them to maintain fluency even when presented with unfamiliar rhythmic and melodic material (Penttinen et al., 2014), underscoring the role of predictive eye-movement strategies.

Recent research on parafoveal processing provides a complementary mechanism for read-ahead behavior. Pan et al. demonstrated that readers extract lexical information from parafoveal regions before saccades occur, suggesting a temporal separation between perceptual intake and motor preparation (Pan et al., 2021). Applied to music reading, parafoveal processing enables musicians to preview upcoming notes, patterns, or structural cues before initiating the eye movement. This mechanism aligns naturally with the proposed bi-temporal model, as saccades support spatial transitions across the score, detecting familiar patterns in the notation and allowing parafoveal processing to anticipate several beats ahead while real-time execution of the music performance takes place.

From a cognitive perspective, these findings of visual scanning and saccades can be situated within the framework of working memory. According to Baddeley's revised model, the phonological loop and visuospatial sketchpad support the short-term retention of symbolic information (Baddeley, 2012); however, their limited capacity necessitates the development of automated retrieval processes for fluent performance. Skilled sight-readers appear to bypass some of these capacity constraints through proceduralized, automatic processing (Ashby et al., 2010), reducing reliance on the prefrontal attentional system. The automaticity hypothesis suggests that repeated sensorimotor coupling of visual notation with motor output strengthens associative networks, allowing musicians to respond reflexively to notated stimuli while simultaneously allocating cognitive resources to higher-order planning and error monitoring (Ashby et al., 2010).

### 3.2 Cognitive processing models of interpreting music notation

In describing how musicians can read and translate music notation, one of the predominant theories guiding music education is Edwin Gordon's audiation framework, a component of Gordon's overall Music Learning Theory (Gordon, 2011). In an earlier outline of his theory, Gordon expressed that “audiation is to music what thought is to language” (Gordon, 1999, p. 42), further clarifying that aural perception is distinct from audiation. Moreover, the act of decoding notation is different from engaging in audiation, as the former does not prescribe an inner sense of hearing.

Psychological research has suggested that highly trained musicians process written music holistically and automatically (Wong and Gauthier, 2010a). Holistic processing on an automatic level is typically associated with facial recognition (Farah et al., 1998) and visual elements unique to a particular area of expertise (Curby and Moerel, 2019). Holistic processing, defined as the failure of selective attention (Curby and Moerel, 2019; Wong and Gauthier, 2010a), shares some aspects with the principles of *Gestalt* perception (Wertheimer, 1912); however, there are some differences between the two processing models. Holistic processing refers to perceiving complex stimuli as integrated wholes, rather than as individual elements. Gestalt principle, on the other hand, describes how visual components may be grouped intuitively to form the impression of a whole. In the context of music, Gestalt is considered by theorists and scholars from the perspective of time (Tenney and Polansky, 1980) and the processing of structures in the musician's mind (Reybrouck, 1996; Terhardt et al., 1987).

When examined in the context of reading notation, Wong and Gauthier found that musicians with higher levels of expertise could distinguish relevant information using brain regions distinct from those of novice music readers (Wong and Gauthier, 2010a). For novice music readers, neural responses were observed in the right fusiform face area (rFFA), the brain region to which holistic processing, such as facial recognition, is attributed (Farah et al., 1998; Maurer et al., 2002; Wong and Gauthier, 2010a). However, for expert music readers, activity in the rFFA was negatively correlated with years of experience, indicating that greater expertise in music reading was associated with holistic processing and automaticity occurring in brain regions other than rFFA (Wong and Gauthier, 2010a). In this study, examples of holistic processing among novice music readers were “strategic” in novice musicians, requiring attention to individual notes for understanding. In contrast, experienced musicians displayed evidence of abilities to identify constituent parts of music notation as well as patterns and sequences similar to that of facial recognition.

Schön et al. found a specific activation focus at the right occipito-temporal junction when pianists read musical notes, a region they likened to a musical analog of the visual word form area for text reading (Schön et al., 2002). This suggests that, through training, the brain develops a specialized area for recognizing visual symbols associated with musical notes. Additionally, musical literacy can induce plastic changes in visual regions, with short-term training in reading music enhancing fusiform gyrus responses to notation (Stewart, 2005a). Such findings indicate that the visual cortex not only detects the shapes of notes but also becomes tuned to the unique patterns of musical symbols in experienced readers.

### 3.3 Systematic review of neuroimaging studies with a focus on music notation

A problem currently present in the literature regarding the neuronal bases of reading music notation is that, apart from a few examples, most brain imaging studies investigating this topic focus on single parameters of music notation reading. This contradicts real-world music performance, which involves many specific components of stimulus-response processing (Gruhn and Rauscher, 2007; Zatorre, 2012). Another issue is the limited research available on prospective planning, an element of performance critical to successful outcomes when reading music from notation. A third concern is the lack of understanding of how musicians can simultaneously operate the extensive neural networks necessary for music performance (Altenmüller and Furuya, 2015).

Imaging studies have yielded significant data on how music affects biological structures of the brain. Such studies encompass a wide range of elements related to music, including listening to music, playing an instrument, interpreting music notation, near and far transfer of skills, and long-term musical training. A large body of research exists on how learning and performing music influence neurobiological structures and promote synaptic plasticity (Proverbio and Sanoubari, 2024), as well as how reading small examples of music notation may be represented hemispherically in the brain (D'Anselmo et al., 2015). These data are essential in their contributions to understanding the neural bases for reading notation. However, there is a scarcity of information regarding how cognitive processes involved in interpreting musical notation operate in the real-world context of a musician simultaneously negotiating numerous actions while prospectively planning for future task operations.

In recent literature, brain imaging studies that incorporate the interpretation of music notation primarily focus on individual parameters, such as pitch or rhythm. While there are instances where pitch and rhythm are combined in music notation stimuli, there may be constraints on complexity, such as using simplified rhythms like quarter and eighth notes. However, the real-world application of reading music for a musician encompasses a vast array of activities that necessarily integrate an expansive neuronal network, while also requiring a high level of attentional focus in the PFC region of the brain. Given the complex integration of tasks necessary to perform music, the neural processes of interpreting music notation need to be evaluated in the context of simultaneous task action alongside multiple sensory stimuli. This sentiment is echoed by Schön and Besson, who argue that “since melody and harmony both contribute to the rhythmic organization of a musical work, and since neither melody nor harmony can be activated without rhythm, the three must be regarded as inseparably linked” (Schön and Besson, 2002, p. 868) and posit that for music to be adequately represented in brain imaging studies, these elements must be integrated.

## 4 Methods and materials

**Figure 1:**
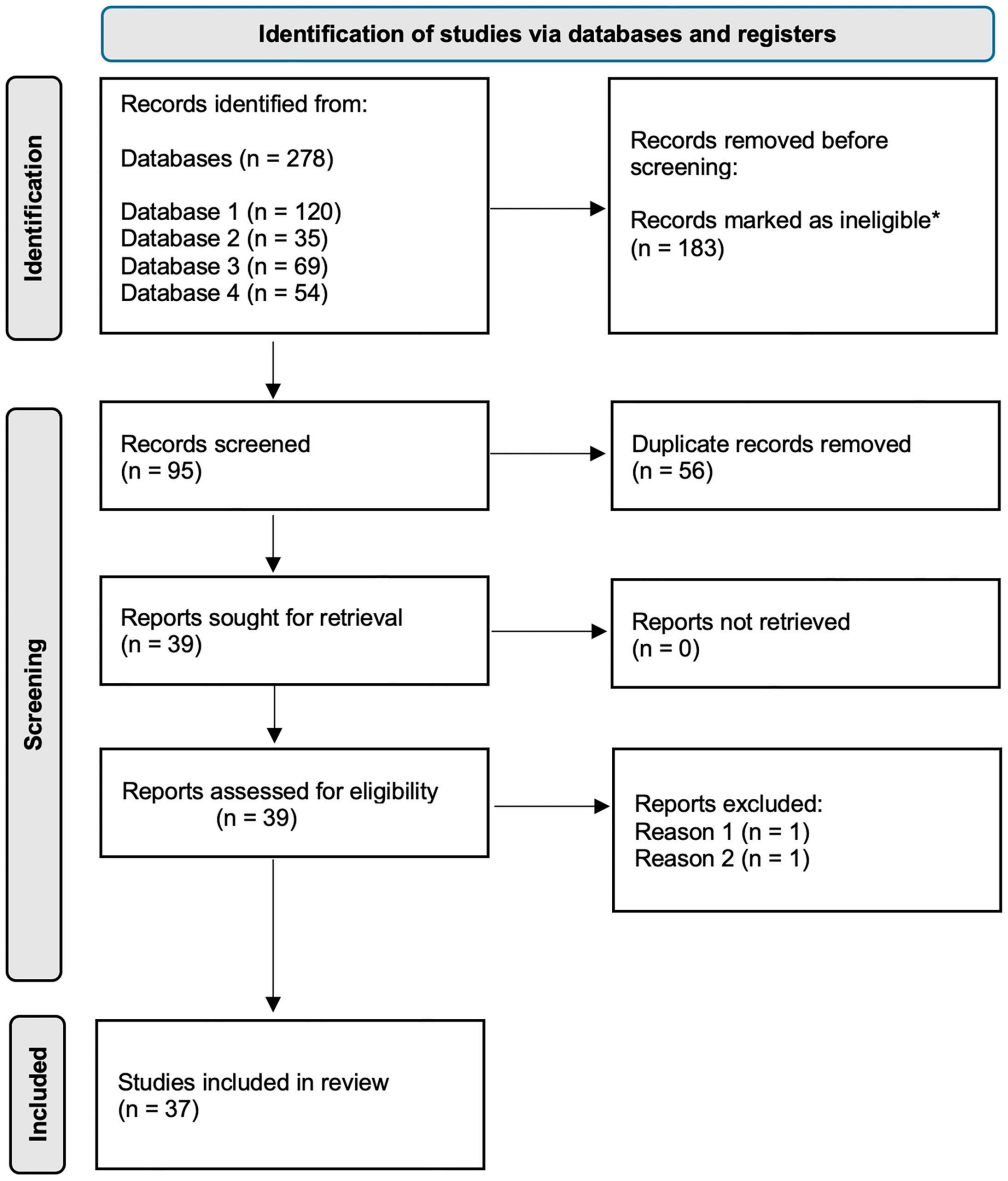
PRISMA 2020 flow diagram. This work is licensed under CC BY 4.0. To view a copy of this license, visit https://creativecommons.org/licenses/by/4.0/. *Reports marked ineligible by the author, not automation tools.

To investigate cognitive processes as they relate to neural bases in interpreting music notation, a review of brain imaging studies of music notation was conducted. This review followed the Preferred Reporting Items for Systematic Reviews and Meta-Analyses (PRISMA 2020) guidelines to ensure transparency and reproducibility in the identification, screening, and inclusion of studies examining the neural correlates of reading music notation (see Figure 1). Employing a systematic approach, defined as “a review of existing research using explicit, accountable, rigorous research methods” (Gough et al., 2017, p. 4), I searched the following electronic databases: Google Scholar, Scopus, Web of Science, and EBSCO, using specific search terms to narrow the results to a high level of relevance. To determine the relevance to the present investigation, information was extracted from the abstracts, with unrelated studies discarded.

The inclusion criteria were as follows: (1) that cognitive functions relating to interpreting music notation were involved in the study; (2) that the tests performed in the studies incorporated responding to the visual stimulus of music notation; (3) that the studies were published in peer-reviewed journals or books (dissertations and theses were not considered); (4) that the studies were of original content, and not, for example, literature reviews of previous studies (i.e., primary studies); and (5) that the results returned were either open access or able to be viewed via institutional subscriptions. The search queries were: (1) fMRI music reading; (2) “fMRI” and “music reading;” (3) “brain imaging” and “music notation;” and (4) “brain imaging” and “music reading.” Analysis was limited to the first 30 results for each search term in each database, resulting in a possible return of 278 results to investigate. After applying the inclusion criteria to each of the 278 articles, 95 listings (34.17%) were identified as meeting the requirements for this review (see Table 1).

**Table 1:** Literature search results: brain imaging and reading music notation.

**Search term**	**Search engine (SE)**	**Results (Total)**	**Results limits**	**Relevant articles**
fMRI music reading	Google Scholar	82,900	30	10
	EBSCO	1,914	30	1
	Scopus	5,821	30	2
	Web of Science	42	30	13
“fMRI” and “music reading”	Google Scholar	853	30	15
	EBSCO	1	1	0
	Scopus	5	5	4
	Web of Science	10	10	8
“brain imaging” and “music notation”	Google Scholar	353	30	12
	EBSCO	2	2	1
	Scopus	10	10	3
	Web of Science	9	9	6
“brain imaging” and “music reading”	Google Scholar	421	30	7
	EBSCO	2	2	2
	Scopus	24	24	6
	Web of Science	5	5	5
Total		92,372	278	95

The total number listed for the column titled relevant articles does not account for duplication of results between the different search engines. Therefore, the real number of relevant articles, novel to each other, is lower than indicated in this table.

An investigation was conducted on publications deemed relevant to this study that summarized the following elements: the brain imaging technique used, the musical parameters under investigation (e.g., pitch, rhythm), and the complexity of these parameters. These analyses served to ground the hypothesis central to this article by providing a perspective on conditions and contexts for musicians who participate in brain imaging studies that incorporate reading music notation.

A narrative synthesis approach was chosen because the studies included in this review demonstrated heterogeneity across imaging modality, task design, and participant expertise. These variations presented limitations for calculating meaningful comparisons, rendering a statistical meta-analysis inappropriate. The narrative synthesis approach preserved the contextual, conceptual, and methodological nuances of each study. This approach facilitated the identification of convergent patterns in neural activation, the evaluation of discrepancies arising from divergent task structures, and the critical examination of ecological validity in existing research on music-notation processing. Furthermore, given that this review aims to develop and refine a theoretical model of bi-temporal cognitive processing, narrative synthesis offered the necessary flexibility to connect empirical results with broader cognitive and neuroscientific frameworks.

## 5 Results

Of the 95 relevant articles identified across the databases, numerous instances of duplication were found. Among these articles, 37 were unique (38.94%), with 22 represented in two or more different databases. The remaining 15 articles appeared in only one database across the 16 possible search iterations (see Table 2).

**Table 2:** Article summary.

**Relevant Article**		
**Represented in two or more databases**	**Represented in a single database**	**Total**
22	15	37

In brain imaging studies on cognitive processing during music notation reading, simple representations of pitch (notes) and duration (rhythm) were commonly used as stimulus materials. This review of the literature focused on pitch and rhythm, either as individual items for study using brain imaging techniques or in simple combinations. Table 3 presents the primary focus and complexity of music notation across the 37 studies, along with the brain imaging techniques employed.

**Table 3:** Review of brain imaging studies involving music notation reading.

**Article No**.	**Author(s)**	**Year**	**Brain imaging technique**	**Primary focus**	**Complexity**	**References**
1	Bouhali, Florence; Valeria Mongelli, Laurent Cohen	2017	Functional magnetic resonance imaging (fMRI)	Pitch and rhythm	Simple: one bar at a time	Bouhali et al., 2017
2	Bouhali, Florence, Valeria Mongelli, Michel Thiebaut de Schotten, and Laurent Cohen	2020	Functional magnetic resonance imaging (fMRI)	Pitch and rhythm	Simple: one bar at a time	Bouhali et al., 2020
3	Brodsky, Warren, Yoav Kessler, Bat-Sheva Rubinstein, Jane Ginsborg, and Avishai Henik	2008	Electromyography (EMG)	Pitch and rhythm	Moderately difficult; up to 16 bars visible to participants	Brodsky et al., 2008
4	Brown, Rachel M, and Virginia B Penhune	2018	Functional magnetic resonance imaging (fMRI)	Pitch and rhythm	Singular notes	Brown and Penhune, 2018
5	Chang, YH.F., Ullén, F. and de Manzano, Ö	2025	Functional magnetic resonance imaging (fMRI)	Pitch	Simple: association with fingers numbered 1 through to 5 with correlations to notes C through to G on a keyboard	Chang et al., 2025
6	de Manzano, Orjan, and Fredrik Ullen	2012	Functional magnetic resonance imaging (fMRI)	Pitch	Simple	de Manzano, Orjan and Ullen, 2012
7	Endestad, Tor, Rolf Inge Godøy, Markus Handal Sneve, Thomas Hagen, Agata Bochynska, and Bruno Laeng	2020	Functional magnetic resonance imaging (fMRI)	Pitch and rhythm	Moderate to difficult	Endestad et al., 2020
8	Giovannelli, Fabio, Simone Rossi, Alessandra Borgheresi, Gioele Gavazzi, Gaetano Zaccara, Maria Pia Viggiano, and Massimo Cincotta	2020	Transcranial magnetic stimulation (TMS), measured by Electromyography (EMG)	Pitch and rhythm	Simple	Giovannelli et al., 2020
9	Hoppe, Christian, Christoph Splittstößer, Klaus Fliessbach, Peter Trautner, Christian E. Elger, and Bernd Weber	2014	Functional magnetic resonance imaging (fMRI)	Pitch and rhythm	Simple: crotchets and Quavers	Hoppe et al., 2014
10	Karagiorgis, Alexandros T., Nikolas Chalas, Maria Karagianni, Georgios Papadelis, Ana B. Vivas, Panagiotis Bamidis, and Evangelos Paraskevopoulos	2021	Electroencephalogram (EEG)	Pitch	Simple	Karagiorgis et al., 2021
11	Kawasaki, A., and Hayashi, N	2022	Transcranial Doppler (TCD) flowmetry	Pitch and rhythm	Simple and familiar: based on *Canon* by Pachabel	Kawasaki and Hayashi, 2022
12	Lee, Horng-Yih, and Sot-Fu Lei	2012	Electroencephalogram (EEG); Horizontal electrooculogram (hEOG); Vertical electrooculogram (vEOG)	Pitch	Simple	Lee and Lei, 2012
13	Lee, Horng-Yih, and Yu-Sin Wang	2011	Electroencephalogram (EEG); Horizontal electrooculogram (hEOG); Vertical electrooculogram (vEOG)	Pitch	Simple	Lee and Wang, 2011
14	Liao, Yin-Chun, Ching-Ju Yang, Hsin-Yen Yu, Chiu-Jung Huang, Tzu-Yi Hong, Wei-Chi Li, Li-Fen Chen, and Jen-Chuen Hsieh	2024	Functional magnetic resonance imaging (fMRI)	Rhythm	Moderate to difficult: improvisation was incorporated alongside notational prompts	Liao et al., 2024a
15	Liao, Yin-Chun, Ching-Ju Yang, Hsin-Yen Yu, Chiu-Jung Huang, Tzu-Yi Hong, Wei-Chi Li, Li-Fen Chen, and Jen-Chuen Hsieh	2024	Functional magnetic resonance imaging (fMRI)	Rhythm	Moderate to difficult: improvisation was incorporated alongside notational prompts	Liao et al., 2024b
16	Lu, Ching-I., Margaret Greenwald, Yung-Yang Lin, and Susan M. Bowyer.	2022	Magnetoencephalography (MEG)	Pitch	Simple to moderate: transposition was involved	Lu et al., 2022
17	Lu, C.-I., Greenwald, M. L., Lin, Y.-Y., and Bowyer, S. M.	2021	Magnetoencephalography (MEG)	Pitch	Simple to moderate: transposition was involved	Lu et al., 2019
18	Lu, Ching-I., Margaret L. Greenwald, Yung-Yang Lin, and Susan M. Bowyer	2019	Magnetoencephalography (MEG)	Pitch	Simple	Lu et al., 2019
19	Meister, Ingo G., Timo Krings, Henrik Foltys, Babak Boroojerdi, Mareike Müller, Rudolf F. Töpper, and Armin K. Thron	2004	Functional magnetic resonance imaging (fMRI)	Pitch	Simple: pitch was used as a control or baseline determinant	Meister et al., 2004
20	Mongelli, Valeria, Stanislas Dehaene, Fabien Vinckier, Isabelle Peretz, Paolo Bartolomeo, and Laurent Cohen	2017	Functional magnetic resonance imaging (fMRI)	Pitch and rhythm	Simple to moderate: up to semiquavers with some ornamentation (trills, mordents); one-bar lengths	Mongelli et al., 2017
21	Nakada, Tsutomu; Fujii, Yukihiko; Suzuki, Kiyotaka; Kwee, Ingrid L	1998	Functional magnetic resonance imaging (fMRI)	Unspecified	The study alluded to general score examples common to piano repertoire literature	Nakada et al., 1998
22	Nichols, Emily S., and Jessica A. Grahn	2016	Electroencephalogram (EEG)	Pitch	Simple	Nichols and Grahn, 2016
23	Paraskevopoulos, Evangelos, Anja Kuchenbuch, Sibylle C. Herholz, Christo Pantev	2014	Magnetoencephalography (MEG)	Pitch	Simple	Paraskevopoulos et al., 2014a
24	Paraskevopoulos, E., Kuchenbuch, A., Herholz, S.C.,... Bamidis, P., Pantev, C	2014	Magnetoencephalography (MEG); Electroencephalogram (EEG)	Pitch	Simple	Paraskevopoulos et al., 2014b
25	Proverbio, Alice M., Matteo Valtolina	2025	Electroencephalogram (EEG); Horizontal electrooculogram (hEOG); Vertical electrooculogram (vEOG)	Rhythm	Moderate to difficult: notation included 280 stimuli involving dotted rhythms (minims, quavers, and semiquavers), semiquavers, and irregular triplet groupings	Proverbio and Valtolina, 2025
26	Proverbio, Alice Mado; Arcuri, Giulia; Maria Pantaleo, Marta; Zani, Alberto; Manfredi, Mirella	2024	Electroencephalogram (EEG); Horizontal electrooculogram (hEOG); Vertical electrooculogram (vEOG); Standardized weighted low-resolution electromagnetic tomography (swLORETA)	Pitch and rhythm	Simple: note recognition involved mi, fa, sol, la, and si	Proverbio et al., 2024
27	Ross, Valerie, Zunairah Haji Murat, Norlida Buniyamin, and Zaini Mohd-Zain	2014	Electroencephalogram (EEG)	Pitch and rhythm	Moderate to difficult: rhythms up to semiquavers	Ross et al., 2014
28	Schön, Daniele, Jean Luc Anton, Muriel Roth, Mireille Besson	2002	Functional magnetic resonance imaging (fMRI)	Pitch	Simple: association with fingers numbered 1 through to 5 with correlations to notes C through to G on a keyboard	Schön et al., 2002
29	Schön, Daniele, and Mireille Besson	2002	Electroencephalogram (EEG)	Pitch and rhythm	Simple	Schön and Besson, 2002
30	Sergent, Justine, Eric Zuck, Sean Terriah, and Brennan MacDonald	1992	Positron emission tomography (PET); Functional magnetic resonance imaging (fMRI)	Unspecified	The study alluded to general score examples common to piano repertoire literature	Sergent et al., 1992
31	Simoens, Veerle L., and Mari Tervaniemi	2013	Electroencephalogram (EEG)	Pitch	Simple	Simoens and Tervaniemi, 2013
32	Stewart, Lauren	2005	Functional magnetic resonance imaging (fMRI)	Pitch	Simple: association with fingers numbered 1 through to 5 with correlations to notes C through to G on a keyboard	Stewart, 2005a,b
33	Stewart, Lauren	2005	Functional magnetic resonance imaging (fMRI)	Pitch	Simple: association with fingers numbered 1 through to 5 with correlations to notes C through to G on a keyboard, graduating toward pure notation	Stewart, 2005a,b
34	Stewart, Lauren, Rik Henson, Knut Kampe, Vincent Walsh, Robert Turner, Uta Firth	2003	Functional magnetic resonance imaging (fMRI)	Pitch	Simple: Association with fingers numbered 1 through to 5 with correlations to notes C through to G on a keyboard, graduating toward pure notation	Stewart et al., 2003
35	Stewart, Lauren, Rik Henson, Knut Kampe, Vincent Walsh, Robert Turner, and Uta Frith	2003	Functional magnetic resonance imaging (fMRI)	Pitch	Simple: association with fingers numbered 1 through to 5 with correlations to notes C through to G on a keyboard, graduating toward pure notation	Stewart et al., 2003a,b
36	Wakita, Masumi.	2016	Functional magnetic resonance imaging (fMRI)	Pitch and rhythm	Simple	Wakita, 2016
37	Wong, Yetta Kwailing, and Isabel Gauthier.	2010	Functional magnetic resonance imaging (fMRI)	Pitch and rhythm	Simple	Wong and Gauthier, 2010a,b

While some of these studies engaged with musical stimuli representing performance contexts as experienced by professional musicians, the simplicity of most of the musical notation calls into question what we really know about brain mapping and cognitive processing when reading music. This simplicity reduces the need for conscious effort for participants with high levels of literacy, and importantly, negates the opportunity to discover neural correlates of simultaneous task function when reading music.

**Figure 2:**
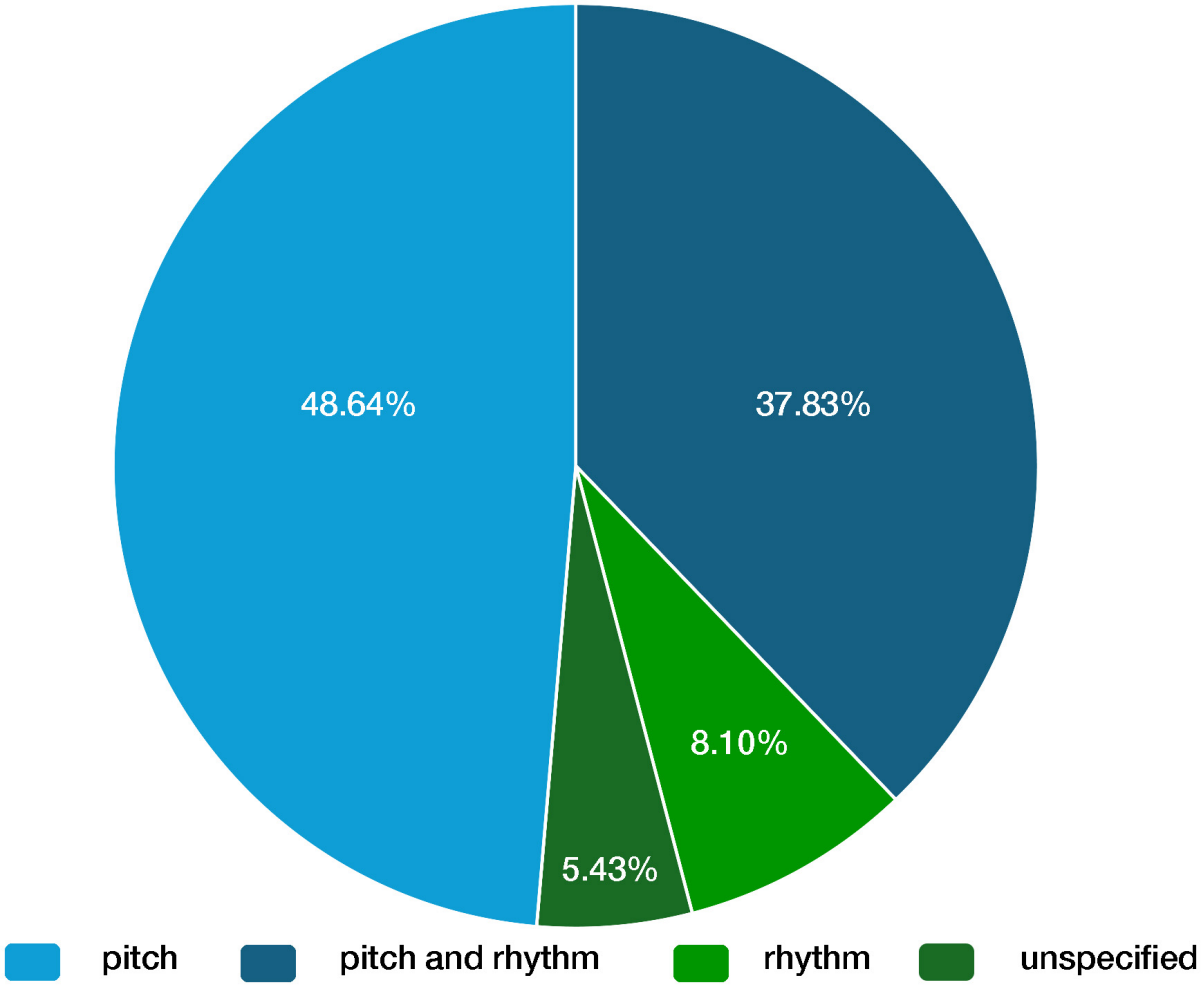
Focus of notational elements across the sample literature.

In terms of content explored in the collection of literature, there were 18 articles focused on pitch (48.64%), 14 articles focused on both pitch and rhythm parameters (37.83%), three articles on rhythm alone (8.10%), and two articles that did not specify a specific focal point. Figure 2 represents the breakdown of article focus across the sample.

**Figure 3:**
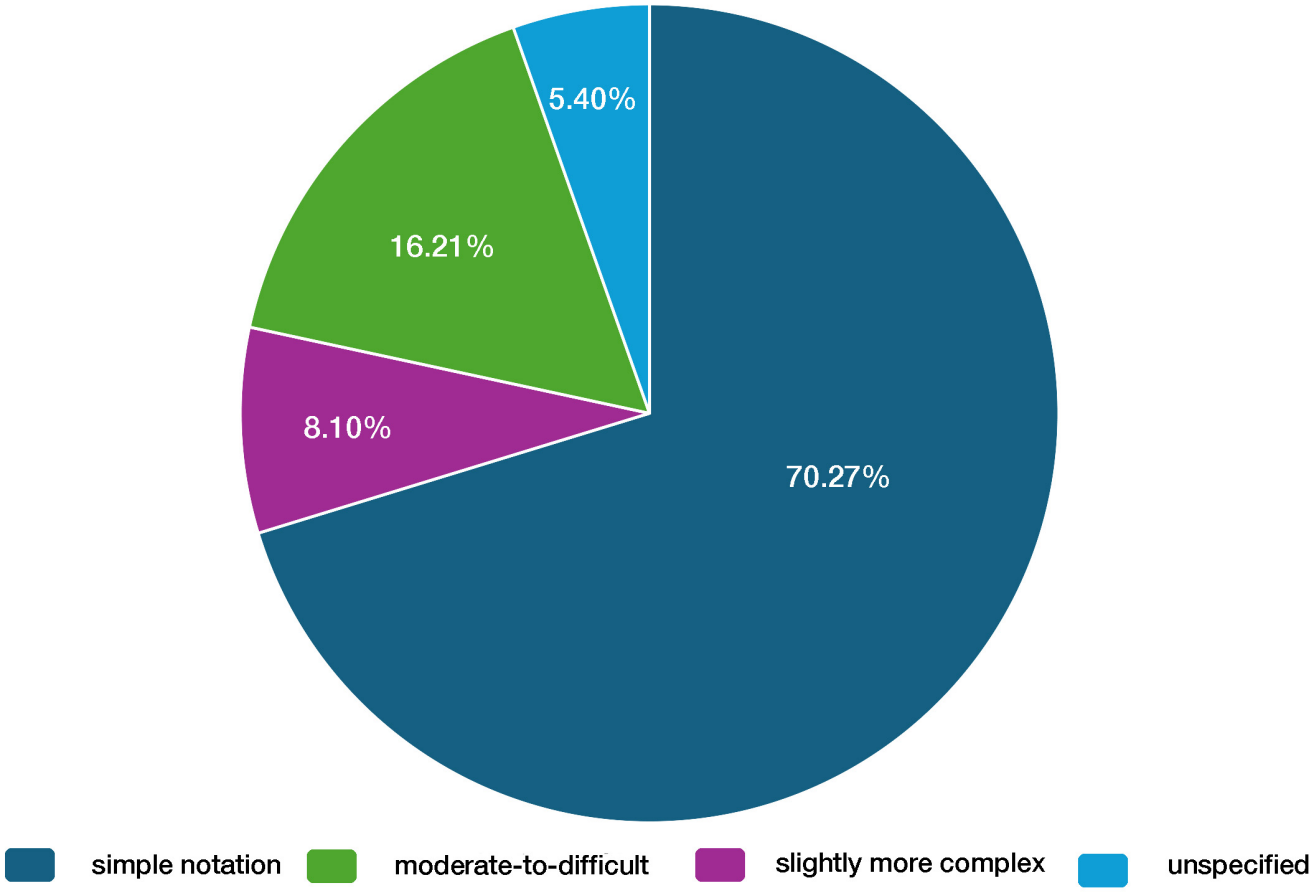
Levels of complexity of music notation used in sample literature.

To summarize the representation of complexity or simplicity in the literature sample, Figure 3 shows that out of the 37 articles examined, 26 (70.27%) used simple music notation as the visual stimuli. In the simple to moderate category, three articles used slightly more complex or layered content to simulate notation stimuli (8.10%). There were six articles that used moderate-to-difficult notational stimuli (16.21%), and two articles referenced piano pieces from the broader repertoire (5.40%).

## 6 Discussion

The convergence of literature from the domains of psychology, neuroscience, and music education highlights the important role of automaticity in instrumental performance and the neural mechanisms underpinning music reading. Overall, the existing literature provides partial but fragmented support for a bi-temporal account of music-notation processing. While behavioral and eye-movement studies are broadly consistent with predictive, dual-pathway processing, neuroimaging evidence remains constrained by limited task complexity and insufficient attention to prospective planning. These limitations do not undermine the model but instead motivate its theoretical necessity, which is more broadly addressed in Section 6.4.

One area that appears to be well-supported in the literature is that, far from being a simple consequence of repetition, automaticity emerges through the interaction of domain-specific cognitive processes and Hebbian-driven plasticity in cortical and subcortical circuits (Jäncke, 2012). Neuroimaging studies demonstrate that reading music notation recruits a distributed network including the visual cortex, superior temporal gyrus, premotor cortex, and supplementary motor areas, which together facilitate the transformation of visual symbols into motor commands and auditory imagery (Stewart et al., 2003b; Schön and Besson, 2002). With sustained practice, musicians transition from a conscious, declarative decoding of notation to the automatic engagement of proceduralized routines, a process associated with strengthened connectivity between the dorsolateral prefrontal cortex, basal ganglia, and cerebellum (Bangert et al., 2006; Zatorre et al., 2007). Taken together, the evidence indicates that music-notation reading recruits distributed visual, auditory, and motor networks that partially overlap with those involved in text reading but diverge in their reliance on predictive audiation and sensorimotor integration, reflecting the performative demands unique to musical notation.

### 6.1 Understanding the limitations of brain imaging studies

There are complexities associated with accurately interpreting brain scans of the human brain. The stochastic nature of activity in biological processes can produce extraneous data that can be analyzed but not necessarily predicted, and may arise due to intrinsic factors, such as normal processes of protein transfer across cell membranes, or extrinsic factors like increased stress hormones resulting from environmental changes (Eling et al., 2019). When measuring dynamic brain activity, such extraneous biological activity, or “noise,” can interfere with the fidelity of the data, potentially resulting in a misrepresentation of neuronal activity. It is therefore crucial that baseline measurements are taken as part of all studies that isolate specific variables for analysis, as well as the resting state of the participants.

Adjacent to the problem of biological noise in brain imaging is the possibility of elevated correlations resulting from generalizations in contemporary brain research analysis. Bandettini points out that a current challenge in referring to statistical mapping structures to interpret analytical findings is that “the maps are not appropriately normalized to multiple comparisons” (Bandettini, 2020, p. 184), which can lead to incorrect analyses when statistical maps are used to form a baseline comparison. Additionally, elevated correlations may be derived when studies are conducted that do not account for independent variables. An example of this is described by Vul et al., in their review of 55 brain imaging studies (53 studies, after two were deemed not to meet the criteria), where they identified non-specific explanations for the conditions under which voxel activations were selected for data analysis (Vul et al., 2009). Specifically, the authors noted that misrepresentations occurred when authors of the 53 studies reported high levels of correlation between activations in specific brain areas and stimuli that provoked responses in emotional, personality, and social cognition categories.

Considering the risks of biological noise interference or unintentional misinterpretation of brain imaging data, it is essential to isolate specific learning tasks or actions when conducting studies of the human mind. Also, because statistical maps informing the interpretation of data may not always be reliable (Bandettini, 2020), responsible practice in obtaining replicable and authentic imaging information must be at the forefront of studies. From there, gradually increasing complexities in music notation stimuli could be possible and may yield results previously undiscovered.

#### 6.1.1 Determining levels of expertise for musician-participants

Another constraint in brain imaging studies is bias, specifically in relation to the level of competency of musicians under study, and how this competency is interpreted. Many studies in this body of literature refer to subjects within their protocols as “trained musicians,” substantiating this by outlining the number of years the participants have taken lessons on their instrument (Brown and Penhune, 2018; Giovannelli et al., 2020; Kawasaki and Hayashi, 2022; Liao et al., 2024b; Lu et al., 2022, 2019; Nichols and Grahn, 2016; Schön et al., 2002; Schön and Besson, 2002; Simoens and Tervaniemi, 2013; Wakita, 2016; Wong and Gauthier, 2010a), or if they perform professionally or study at a conservatoire (Bouhali et al., 2017, 2020; Brodsky et al., 2008; de Manzano and Ullén, 2012; Endestad et al., 2020; Hoppe et al., 2014; Lee and Lei, 2012; Liao et al., 2024a,b; Meister et al., 2004; Mongelli et al., 2017; Paraskevopoulos et al., 2014b; Proverbio and Valtolina, 2025; Ross et al., 2013; Simoens and Tervaniemi, 2013; Stewart, 2005a,b). If a study focuses on the entrainment aspects of novel musical information for non-musicians (Stewart et al., 2003a), a restriction on the time spent previously learning an instrument may be stated (Chang et al., 2025; Karagiorgis et al., 2021; Stewart et al., 2003b) or a declaration may be made that the participants had not received formal training prior to the study (Lee and Wang, 2011; Paraskevopoulos et al., 2014a).

However, even at the professional level, there is a variance in skill competencies, which is why musicians choose to specialize in certain areas. Furthermore, conservatorium-trained musicians who become career musicians in orchestras may refine their focus to either traditional classical music, playing in a philharmonic or symphony orchestra. If they specialize in contemporary classical music, they are likely to play in smaller, chamber ensembles, often with more challenging and soloistic repertoire. The distinction between even just these two styles of music—two styles out of several hundred—raises a critical question about musical language and what a musician may be familiar with in terms of patterns and paradigms. Some studies investigating how musicians interpret music notation claim to use lesser-known etudes or works composed by specialists as an unknown stimulus for musician participants, yet the underlying language within such music is steeped in predictability. When adhering to a style associated with traditional forms of classical music, detectable patterns and compositional characteristics can be distilled into easily identifiable scale and arpeggio-based fragments. Altenmüller and Furuya describe this phenomenon as musicians having a “similar acculturation due to the canonical nature of their training” (Altenmüller and Furuya, 2015, p. 4). Therefore, using music notation that displays stylistic characteristics typical of the classical genre does not provide a novel stimulus of music. This crucial point potentially circumvents the aims of studies that investigate the cognitive processing of unrehearsed musical material as a central focus. Instead, data derived from a study that relies on classical-archetypal notational material will likely measure the identification of deeply familiar patterns, forged from a strong procedural memory that has been consolidated over years of practice, rather than responses to “novel” musical stimuli.

#### 6.1.2 Different levels of musical ability and implications for data reliability

The studies in this literature review offered varying levels of detail regarding the musician-participants' levels of experience and competencies. While some studies included detailed supplementary material with fulsome information about the participants validating their expertise (Proverbio and Valtolina, 2025), others considered musical training in excess of one year to be sufficient for a participant to be categorized as a musician (Nichols and Grahn, 2016). In a study using EEG, hEOG, and vEOG, Proverbio and Valtolina acknowledged the limitations of available musical stimuli for advanced musicians (Proverbio and Valtolina, 2025). Discounting the use of rhythmic tests, such as the *Montreal Battery of Evaluation of Musical Abilities*, in baseline testing because they were too simple for the experienced musician participants, the authors opted to incorporate more complex notational stimuli in the tests, aligning more closely with real-world applications of performing from notation for highly trained musicians.

Stewart et al. presented a robust publication in which musically naïve participants were under study for evidence of skill development (Stewart et al., 2003b). To quantify the level of performance target, participants were given music notation and theory stimuli at a Grade 1 level (UK: Associated Board of the Royal Schools of Music). Because participant progress was not uniform, some extra tuition was required to achieve the baseline level of proficiency needed to complete the testing. Another example of considered attention to the variable of musical competency is seen in Wong and Gauthier's study. Here, the authors acknowledge that the automatic holistic processing of notation observed in musician participants was potentially representative of “an increased tendency to process relative positions of notes in music sequences, as one becomes more proficient with musical notation” (Wong and Gauthier, 2010a, p. 549), noting the correlation of years of musical training and fluency in reading notation. Furthermore, Wong and Gauthier accurately pointed out in this study that levels of fluency in reading notation are variable, even within populations of expert musicians.

### 6.2 The case for real-world replication in brain imaging studies

There are many constituent parts to music notation that extend beyond these two elements of pitch and duration, which were highly represented in Table 3. A piece of music will commonly include elements such as tonality (the key to which the piece belongs), dynamics (volume), and articulation (different ways in which a note may be accented, shortened, or stressed). Furthermore, there may be significant modulations throughout a piece, such as tempo changes (speeding up or slowing down as indicated by the instructions included on the sheet music), key signature changes (alterations to not only how many sharps or flats the musician needs to remember but also which scale or tonality they are working in), meter (the time signature that instructs the musician on how to count the beats), the structure (repeat signs and other instructions that inform the musician whether they are to repeat certain sections), and clef changes (the symbol at the beginning of a piece of music that informs the musician how to interpret the pitch placement on the five-lined staff). Table 4 provides a non-exhaustive overview of the parameters a musician simultaneously manages when performing.

**Table 4:** Parameters of music notation: examples.

**Parameter**	**Description**	**Example**
Rhythm	Rhythms as represented in notation indicate the duration of notes	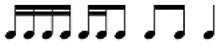
Notes	Notes represent height and/or depth of pitch	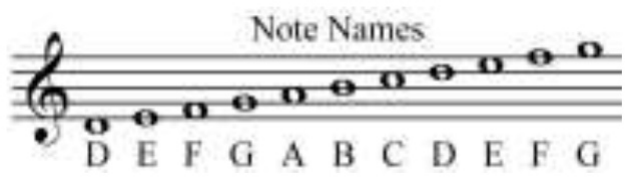
Tonality	The tonality within a piece of music relates to which scale the music is written in. Tonality can be transient and move from one to another as rapidly as the composer desires. This is represented by either key signature changes or accidentals within a piece	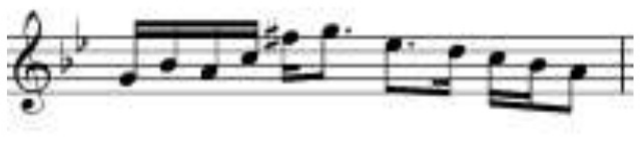
Dynamics	Dynamics represent the volume within a piece of music. These dynamics are often modulatory throughout. The example to the right (forte) indicates a strong, or loud dynamic	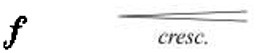
Articulation	Articulation symbols in music indicate different stresses, lengths, and accents upon specified notes	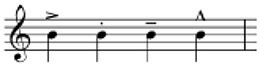
Tempo	Tempo is the speed at which music is played. Notation within a piece of music may include a specific tempo marking, a tempo range, and/or modulating tempi (i.e, speeding up or slowing down)	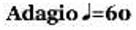
Key Signature	A key signature indicates whether the musician is to play or sing specified sharps or flats. A key signature represents notes that relate to different scales or tonalities. Key signatures can be changed within a piece of music	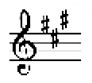
Accidentals	Accidentals in a piece of music are represented by sharps, flats, naturals, double sharps, and double flats. This specifies the pitch that the musician is to play	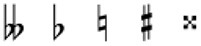
Meter	In music notation, the meter instructs the musician how to count the beats in each bar	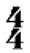
Structure	In music notation, there may be instructions for musicians concerning a repeat of certain sections (e.g., repeat bars), how many times a section is to be repeated, and whether or not they are to skip specified bars on a second play-through (e.g., da capo al fine).	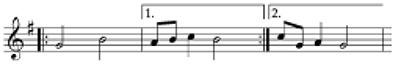
Clef	A clef indicates to a musician how to interpret the pitch indicated on the five-lined staff upon which music is written. Different clefs orientate the pitches in at different points	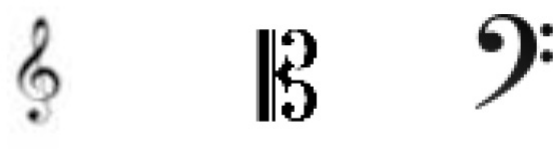

**Figure 4:**
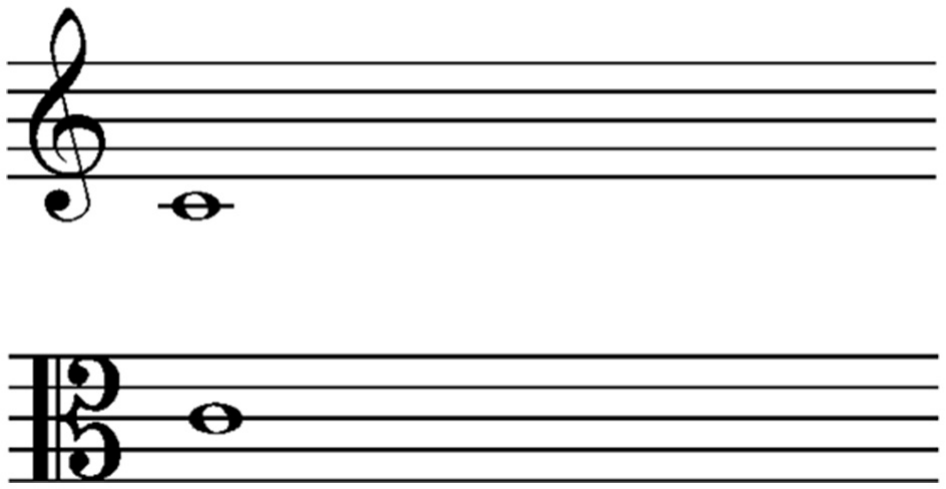
Example of Different Clefs in Music. The treble clef (**top**) instructs the musician how to interpret the note placement. The note to the right of the treble clef is C4. The alto clef (**bottom**) has repositioned the range of notes that the musician now interprets when reading the sheet music. The note to the right of the alto clef is also a C4.

Clef changes are particularly interesting because a musician who is switching between, say, a treble clef and an alto clef, will need to reconfigure their mental positioning of the notes they are looking at. In such an example, what visually presents as a B4 in treble clef becomes a seventh lower in the alto clef (C4). Figure 4 demonstrates the effect of a clef change from treble to alto: the same note is represented in two vastly different ways.

When reading notation, the musician retains contextual information that determines how to play a piece (i.e., which clef is in use or which key signature to apply) through executive functioning. When a piece changes clef, key, dynamics, articulation, or other elements, the musician adapts to the new paradigm. Such shifts can only be achieved with success through prospective planning and an accompanying degree of automaticity that can sufficiently process the changing visual stimuli.

### 6.3 Comparing the interpretation of music notation and the written word

Reading music to a high level involves not only a deep knowledge of the visual representations of notation but also a near-immediate processing of the musical material. Interpreting music from notation is a complex task that is thought to be learned through explicit teaching (Hébert and Cuddy, 2006). A prevailing difference between the interpretation of musical notation and written text is that notation requires the decoding of multiple events and instructions that co-occur, whereas reading words on a page is operationalized in a linear, sequential manner (Sloboda, 2004). Furthermore, reading music notation requires a musician to perform what they see, assessing the aural output in real-time while simultaneously reading notation that has yet to be played. According to Wöllner and Williamon, musicians can construct “sonic images,” which allows for the anticipation of aural stimuli, whether or not auditory feedback is available (Wöllner and Williamon, 2007). Musicians with high levels of expertise conceptualize these sonic images, and when in the act of reading notation, this process is commonly referred to as *audiation*, that is, the “ability to hear and give meaning to music when sound is not physically present or may never have been physically present” (Gordon, 2011, p. 10). Audiation is a cornerstone of accurate musical performance, and its use contributes to the prospective planning that is necessary when playing an instrument or singing.

### 6.4 Introducing the theory of bi-temporal cognitive processing in reading music notation

**Figure 5:**
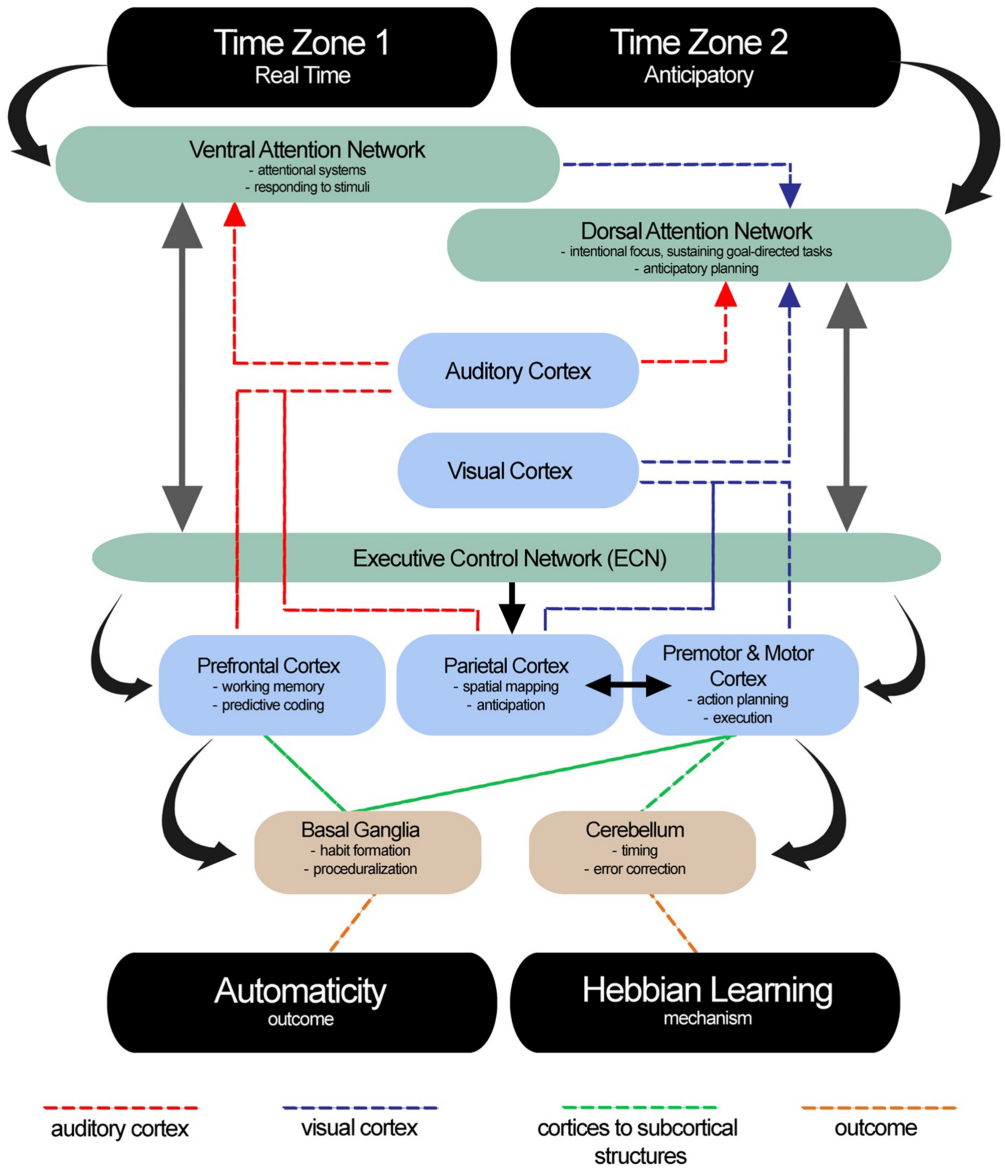
Bi-Temporal Cognitive Processing Theory for Music Notation Reading. Automaticity in music performance reflects the integration of cortical and subcortical systems operating in dual temporal domains (Zatorre et al., 2007). Premotor-motor circuits mediate real-time execution (Scott, 2004; Shenoy et al., 2013; Porter and Lemon, 1995), while anticipatory planning engages prefrontal and parietal regions (Miller and Cohen, 2001); these processes converge in the basal ganglia and cerebellum (Ashby et al., 2010), where Hebbian plasticity consolidates proceduralization and error correction, enabling fluent, predictive performance (Wolpert et al., 1998). This graphic representation is the author's own work.

Several factors operationalize the neural basis of reading music notation. First, the capacity for musicians to enact prospective and retrospective planning simultaneously (Tsao et al., 2022) enables them to perform in real time while reading ahead in the score. This act engages working memory and predictive coding mechanisms in the prefrontal and parietal cortices (Waters et al., 1998; Palmer and van de Sande, 1993). Second, purposeful, repetitive practice over a long period will lead to Hebbian learning as the musician develops their skill set. Maintaining this practice will eventually result in automaticity. Thus, Hebbian learning and the emergence of procedural automaticity provide the neural conditions under which present-focused execution can proceed in parallel with future-oriented prediction, resolving the apparent multitasking paradox in skilled music reading. Third, the act of reading music requires the musician to read ahead and audiate the notation while concurrently performing notation read only moments earlier. This means that a bi-temporal cognitive processing paradigm is central to the success of musical performances: the musician operates in two time zones, producing a single musical outcome. The proposed time zones as seen in Figure 5 reflect functional, temporal orientation rather than discrete neural substrates.

Figure 5 illustrates how automaticity in music performance emerges from distributed cortical and subcortical interactions that operate simultaneously in two cognitive time zones. The visual cortex initiates processing by decoding notation, which is then projected into distinct but overlapping functional streams. In Time Zone 1, information is initially processed through the ventral attention network before moving through the premotor and motor cortices, supporting real-time action response in performance. In Time Zone 2, the dorsal attention network is activated along with the prefrontal and parietal cortices, which engage working memory, predictive coding, and spatial anticipation. This network distribution enables musicians to plan prospective actions while monitoring recent performance events. These cortical processes converge on subcortical structures: the basal ganglia, which support habit formation and proceduralization, and the cerebellum, which refines timing and error correction. Through repetitive, purposeful practice, Hebbian plasticity strengthens these networks, consolidating the mapping of visual symbols onto motor and auditory representations. The outcome is automaticity in reading music notation, characterized by the ability to perform fluently in real time while anticipating future musical events. This bi-temporal framework provides a neural basis for the dual processing demands unique to skilled music performance and the interpretation of notation.

## 7 Conclusion

This theoretical article follows a line of inquiry into the underpinnings of interpreting musical notation within the context of authentic application, the differences between reading music and the written word, the concept of bi-temporal focus and engagement, and Hebbian learning as a basis for understanding automaticity in music performance. A systematic review of the literature examining standard practices and contexts for brain imaging studies provided an essential perspective for discussion. Here, the dissonances between real-world music performance contexts and the musical stimuli found in brain imaging studies were outlined. Connections were drawn between the distinct elements of reading music, aiming to consolidate an understanding of how the musical mind interprets notation.

The theory central to this article is a considered evaluation of cognitive processes that frame the neural basis of interpreting notation in the context of musical performance—a cognitive framework for reading music that hypothesizes the simultaneous dual processing of notational stimuli across two time zones. The pedagogical benefits of deliberate practice as a facilitator of transitioning from controlled to automatic notation processing are significant; understanding this phenomenon in depth might inform future teaching practices. In the fields of psychology and neuroscience, this theory has the potential to guide future research into the neural correlates of reading musical notation, as well as other studies investigating temporally driven cognition.

### 7.1 Testing the theory: recommendations for future study

To interrogate the plausibility of the theory, brain imaging studies that combine the many facets of music performance along with challenging repertoire as the visual stimuli might reveal new perspectives on reading notation. A framework for a study could include secondary tasks that involve temporal activities, measuring neural structures for time, prospective, and retrospective planning in contexts other than music. Comparing these data with scans of musicians in the act of performance might provide evidence supporting the argument that the cognitive processing of music occurs in a bi-temporal fashion. To test the bi-temporal model directly, future studies must be capable of isolating simultaneous execution and prediction rather than treating music reading as a unidimensional decoding task.

Another aspect of this theory to explore is that of Hebbian learning and automaticity. Isolating these factors in a longitudinal study before conducting an intervention study involving bi-temporal cognitive processing activities may be important in ascertaining the skill level required to test the theory authentically. Obtaining these data could define what constitutes a “trained” musician, directing evaluations away from the number of years spent learning an instrument or a working knowledge of notes and musical performance, toward fundamental skills of automaticity resulting from deliberate and repetitive practice.

## Data Availability

The original contributions presented in the study are included in the article/supplementary material, further inquiries can be directed to the corresponding author.

## Footnotes

1 Music notation is not limited only to a visual Western traditional classical music model. For example, sheet music is available for blind people through Braille systems, which provides comprehensive information for the musician performing the music. However, for the purposes of this article, the focus will be on the visual interpretation of notation that is widely used through the media of either printed sheet music or PDF document viewed on a computer or tablet.
